# Supplementary material for: Deciphering intercellular communication in the cerebellar external granule layer: Insights into non-classical connections in neural development
Source: PLoS One. 2026 Jun 25;21(6):e0352088. doi: 10.1371/journal.pone.0352088 (PMC13298772; doi:10.1371/journal.pone.0352088)
Supplement: Attachment — Submitted filename: full-revision_j.pdf [file pone.0352088.s006.pdf]

Full Revision

Manuscript number: RC-2025-03159
Corresponding author(s): Chiara, Zurzolo

[Please use this template only if the submitted manuscript should be considered by the affiliate journal as a full revision in response to the points raised by the reviewers.

If you wish to submit a preliminary revision with a revision plan, please use our "[Revision Plan]" template. ***It is important to use the appropriate template to clearly inform the editors of your intentions.****]*

1. General Statements [optional]

This section is optional. Insert here any general statements you wish to make about the goal of the study or about the reviews.

We thank the reviewers for their thorough and constructive feedback. We have carefully revised the manuscript to address all comments. Substantial clarifications and figure updates have been made to improve the precision, transparency, and readability of our study. To avoid overstatement, we specified that the structures we observed are 'putative TNT-like structures'. For the same purpose, we have changed the title to 'Deciphering Intercellular Communication in the Cerebellar External Granule Layer: Insights into Non-Classical Connections in Neural Development'. The revised version includes restructured sections in the Results and Methods, clearer quantifications, and enhanced figure annotations. We believe these revisions have considerably strengthened the manuscript.

2. Point-by-point description of the revisions

This section is mandatory. Please insert a point-by-point reply describing the revisions that were already carried out and included in the transferred manuscript.

Reviewer #1

1. Clarified that CBs are more abundant in the oEGL and their presence in the iEGL was emphasized to show that divisions occur there as well. Title of section modified accordingly.
2. Retained Figures 1–2 in the main text but strengthened the explanation of their purpose and relationship to Figure 3. Added mention of potential EdU pulse–chase experiments in

the Discussion. Added a schematic (Figure S1) that links our previous study (Cordero et al., 2023) to this new study
3. Added citation to Altman & Bayer for EGL layer proliferation studies.
4. Enlarged schematic illustrations and adjusted colors for GFP/anillin.
5. Revised results to explicitly reference that TAG-1 and Ki67 were used in a CNN-based classifier to define the iEGL mask.

Reviewer #2

1. Clarified the use of Ki67 nuclear patterns (not intensity) to define cell-cycle states; renamed classes accordingly ($Ki67^{mitotic}$, $Ki67^{interphase}$, $Ki67^{G0}$).
2. Clarified that Cytbow labeling identifies *putatively* clonally related cells; discussed stochasticity, sparse labeling, and color overlap limitations.
3. Explained the use of CNN-based segmentation for iEGL delineation and nuclei quantification.
4. Clarified rationale for comparing CB and mitotic-cell frequencies (contextualizing 3.76% from previous study), and added Figure S1 for a clarity
5. Added absolute numbers (mice, images, cells) to Results and figure legends.
6. Clarified imaging acquisition and linear display adjustments.
7. Acknowledged limitations of cytoplasmic labeling and potential underestimation of TNT-like structures.
8. Clarified segmentation resolution and added iEGL mask visualization to Figure 1.

Reviewer #3

1. Added absolute counts for non-normalized quantified parameters.
2. Clarified image processing to confirm no overexposure; noted linear adjustments.
3. Addressed fluorophore limitations (cytoplasmic vs. membrane-bound) in Discussion.
4. Clarified the boundary definition of the iEGL–oEGL and added corresponding figure panels.
5. Clarified purpose and interpretation of Figure 1C and Figure 2B.
6. Added the anatomical location of observations (medial vermis, lobule VIII–IX).
7. Revised Discussion to explicitly acknowledge that the TNT-like connections remain “putative” and require functional validation.
