# Supplementary material for: Deciphering intercellular communication in the cerebellar external granule layer: Insights into non-classical connections in neural development
Source: PLoS One. 2026 Jun 25;21(6):e0352088. doi: 10.1371/journal.pone.0352088 (PMC13298772; doi:10.1371/journal.pone.0352088)
Supplement: Attachment — Submitted filename: report_10.1101+2025.08.11.669634.pdf [file pone.0352088.s005.pdf]

ScreenIT Report

This is an automated report for 10.1101+2025.08.11.669634.pdf. This report was solicited by the PLOS One editorial team and provided by ScreenIT.

ScreenIT is an independent group of scientists developing automated tools that analyze academic papers. A set of automated tools screened your submitted manuscript and provided the report below. Each tool was created by your academic colleagues with the goal of helping authors.

The tools look for factors that are important for transparency, rigor and reproducibility, and we hope that the report might help you to improve reporting in your manuscript. Within the report you will find links to more information about the items that the tools check. These links include helpful papers, websites, or videos that explain why the item is important.

While our screening tools aim to improve and maintain quality standards they may, on occasion, miss nuances specific to your study type or flag something incorrectly. Each tool has limitations that are described on the ScreenIT website. The tools screen the main file for the paper; they are not able to screen supplements stored in separate files.

Please note that the Academic Editor had access to these comments while making a decision on your manuscript. The Academic Editor may ask that issues flagged in this report be addressed.

If you would like to provide feedback on the ScreenIT tool, please email the team at ScreenIt@bih-charite.de. If you have questions or concerns about the review process, please contact the PLOS One office at plosone@plos.org.

How to read the report:

- no action required

? - review for potential issues

! - a critical issue was found by a tool

n/a - may not be applicable to your paper

- more information about this item can be obtained using this link

Rigor

	Ethics	IACUC: "The care and handling of the animals prior or during the experimental procedures followed European Union rules and were approved by the French Animal Protection Committee and the local Ethics Committees."<br>IRB: "The care and handling of the animals prior or during the experimental procedures followed European Union rules and were approved by the French Animal Protection Committee and the local Ethics Committees."<br>Euthanasia Agents: "Briefly, pups were decapitated and the brain dissected out in oxygenated, ice-cold complete HBSS (cHBSS) buffer."
?	Inclusion and Exclusion Criteria	Not detected. Please specify any pre-defined criteria used to determine whether participants, animals or samples were eligible for the study, or exclude participants, animals or samples from the study.
?	Flow charts and attrition	We did not find a study flow chart of excluded observations. We strongly recommend using flow charts because they provide an overview of the study design and more information about attrition. If you included a study flow chart in your supplemental files, we apologize for missing this. Our tool is not able to screen separate supplemental files.<br><br>Sentence about attrition: not detected. Please provide information about the drop-out of subjects, or loss of animals or samples. This could be done using a study flow chart, or described in the text.
	Sex as a biological variable	"To generate experimental animals, we crossed homozygous CAG-Cytbow+/+ males with Nestin-CreERT2+/ females (Lagace et al., 2007) to obtain Nestin-CreERT2+/-; CAG-Cytbow+/offspring."
	Subject Demographics	Age: "C57BL/6 mice at P7 were used for the quantification of CBs and mitotic cells in the iEGL."
	Randomization	

✓		"All image tiles were rescaled before training and split randomly into training (80%) and test (20%) sets."
?	Blinding	Not detected. Please specify whether blinding was used at various phases of the experiment (e.g., blinding of patients, caregivers, outcome assessments, data analysis).
?	Power Analysis	Not detected. Please state how you determined the sample size for the study. If you performed a power or sample size calculation, please provide details.
✓	Replication	"Cordero Cervantes et al., 2023) could be fully accounted for by CBs, we replicated our previous analysis by quantifying mitotic cells (prophase, prometaphase, metaphase, anaphase, and telophase) and Aurora B kinasepositive CBs in the iEGL using immunofluorescence, and compared these values with those previously obtained using an ssSEM-based approach (Fig. 1D, S1A, S1B)."

Generated by Barzooka (flow charts), SciScore (everything else). SciScore only screens the text of the methods section; we apologize if the tool has missed items reported elsewhere in the paper or supplements.

Resources

Persistent unique identifiers for research resources, RRIDs, can be used to track these key resources though the literature. They are requested or required in many journals, but even if your journal does not require them, we strongly encourage the use of these for the readers of papers who wish to easily track down the key materials like antibodies, software tools, or mice.

Antibodies	"First, slices were immuno-labelled with the rabbit antiAurora B primary antibody before running a second IHC with the other antibodies including the rabbit anti-Ki67 antibody."	? antiAurora B suggested: None. Please add an RRID to help others identify this resource.
Antibodies	"Primary antibodies used were: rabbit anti-	? anti-Aurora B kinase (Abcam , ab2254 suggested: None.

	Aurora B kinase (Abcam, ab2254, 1:500), rabbit anti-Ki67 (Abcam, ab15580, 1:200), goat anti-TAG1 (rndsystems, AF4439, 1:200), mouse anti-citron kinase (BD Biosciences, 611376, 1:200), goat anti-anillin (Abcam, ab5910, 1:50), rabbit anti-anillin (Bethyl laborories, A301-406A, 1:500), chicken anti-GFP (Aves Lab, GFP-1010, 1:1000), goat anti-tdTomato (Sicgen, AB8181, 1:200), rat anti-PH3 (Abcam, ab10543, 1:200)."	Please add an RRID to help others identify this resource.<br>anti-Ki67 suggested: None. Please add an RRID to help others identify this resource.<br>anti-TAG1 suggested: None. Please add an RRID to help others identify this resource.<br>AF4439 suggested: None. Please add an RRID to help others identify this resource.<br>anti-anillin (Abcam , suggested: None. Please add an RRID to help others identify this resource.<br>anti-anillin suggested: None. Please add an RRID to help others identify this resource.<br>A301-406A suggested: None. Please add an RRID to help others identify this resource.<br>GFP-1010 suggested: None. Please add an RRID to help others identify this resource.<br>anti-tdTomato suggested: None. Please add an RRID to help others identify this resource.<br>AB8181 suggested: None. Please add an RRID to help others identify this resource.<br>anti-PH3 suggested: None. Please add an RRID to help others identify this resource.
Antibodies	"Results Cytokinetic bridges (CBs) are present throughout the EGL but less frequent in the iEGL To assess the presence of mitotic cells and CBs in the EGL in P7	anti-Aurora B kinase suggested: None. Please add an RRID to help others identify this resource.

	mice, we labeled mitotic cells with an anti-Ki67 antibody and CBs with an anti-Aurora B kinase antibody (Fig. 1A, S1A)."	
Antibodies	"To further differentiate stable IBs from CBs, we labelled midbodycontaining bridges using an anti-citron kinase antibody, which marks midbodies in CBs but not mature IBs (Price et al., 2023) and an anti-anillin antibody, previously reported to label IBs in the Drosophila larval brain (Haglund et al., 2011), (Fig. 2C, S1B)."	? anti-citron kinase suggested: None. Please add an RRID to help others identify this resource.
Antibodies	"Anti-citron kinase and anti-Cep55 antibodies were not targeted because their punctate staining made specific labelling difficult to distinguish from background."	? anti-Cep55 suggested: None. Please add an RRID to help others identify this resource.
Antibodies	"The tdTomato signal was enhanced using an anti-tdTomato antibody, while eYFP was amplified using an anti-GFP antibody which also detected mTurquoise2, and to a lesser extent eBFP2 (Fig."	? anti-GFP suggested: None. Please add an RRID to help others identify this resource.

Experimental Models: Organisms/ Strains	"C57BL/6 mice at P7 were used for the quantification of CBs and mitotic cells in the iEGL."	C57BL/6 suggested: RRID:IMSR_JAX:000664. Please make sure this RRID suggestion is accurate before including in your manuscript.
Experimental Models: Organisms/ Strains	"To generate experimental animals, we crossed homozygous CAG-Cytbow+/+ males with Nestin-CreERT2+/ females (Lagace et al., 2007) to obtain Nestin-CreERT2+/-; CAG-Cytbow+/ offspring."	CAG-Cytbow+/+ suggested: None. Please add an RRID to help others identify this resource.
Experimental Models: Organisms/ Strains	"To increase the probability of combinatorial fluorescent protein expression of in a Cre-dependent manner, these Nestin-CreERT2+/-; CAG-Cytbow+/females were backcrossed with CAGCytbow+/ + males to generate Nestin-CreERT2+/-; CAG-Cytbow+/ +experimental animals."	Nestin-CreERT2+/-; CAG-Cytbow+/+experimental suggested: None. Please add an RRID to help others identify this resource.
Experimental Models: Organisms/ Strains	"Tamoxifen injection 114 To sparsely label neural cells, Nestin-CreERT2+/-; CAG-Cytbow+/+ newborn mice received subcutaneous injection of 25 L of tamoxifen	Nestin-CreERT2+/-; CAG-Cytbow+/+ suggested: None. Please add an RRID to help others identify this resource.

	(234-118-0, Merck) diluted in corn oil once per day at P0, P1, P2 and P3 at a concentration of 5 mg/mL."	
Experimental Models: Organisms/ Strains	"Nestin-CreERT2+/ mice in which tamoxifen induces CRE expression only in neural progenitors were crossed with Cytbow+/+ mice (Loulier et al., 2014) in which mTurquoise, eYFP and tdTomato are stochastically expressed following CRE-mediated recombination."	Nestin-CreERT2+/mice suggested: None. Please add an RRID to help others identify this resource.
Recombinant DNA	"Plasmid Coding sequence of pCAG-Dendra2-E2A-EGFP-Cep55 construct was cloned from pDESTDendra2-E2A-EGFP-Cep55 construct (used in Korenkova et al., 2024) into the pCAG-GFP plasmid from Addgene (Plasmid #11150)"	pDESTDendra2-E2A-EGFP-Cep55 suggested: None. Please add an RRID to help others identify this resource.<br>pCAG-GFP suggested: None. Please add an RRID to help others identify this resource.
Recombinant DNA	"Microscopy and image analysis Imaging Images of cerebellar sections were acquired on a Zeiss LSM900 confocal microscope, except for a subset of samples electroporated with the pCAG-Dendra2-E2A-EGFP-Cep55	pCAG-Dendra2-E2A-EGFP-Cep55 suggested: None. Please add an RRID to help others identify this resource.

	plasmid which were imaged on a Nikon Ti2E Spinning disk microscope."	
Recombinant DNA	"P5 pups were electroporated to transfer a pCAG-Dendra2-E2A-GFP-Cep55 plasmid in GCs progenitors (Fig."	pCAG-Dendra2-E2A-GFP-Cep55 suggested: None. Please add an RRID to help others identify this resource.
Software and Algorithms	"4 were conducted across the entire cerebellum, although labelled cells were predominantly located in lobule IX and X. 2D images were processed with Fiji (ImageJ)."	Fiji suggested: None. Please add an RRID to help others identify this resource.<br>ImageJ suggested: ImageJ, RRID:SCR_003070. Please make sure this RRID suggestion is accurate before including in your manuscript.
Software and Algorithms	"The total number of labelled cells was counted manually using the Spots function in Imaris."	Imaris suggested: None. Please add an RRID to help others identify this resource.
Software and Algorithms	"Statistical analysis was performed using Microsoft Excel and GraphPad Prism."	Microsoft Excel suggested: Microsoft Excel, RRID:SCR_016137. Please make sure this RRID suggestion is accurate before including in your manuscript.<br>GraphPad Prism suggested: None. Please add an RRID to help others identify this resource.

Generated by SciScore.
SciScore only screens the text of the methods section; we apologize if the tool has missed items reported elsewhere in the paper or supplements.

Transparency

?	Open code	If you wrote code to analyze your data, please consider sharing the code in a public repository. This makes it easier for others to

		reproduce your analyses, and may also aid others seeking to analyze similar datasets.
?	Open data	ℹ If permitted, sharing data on a public repository with a persistent identifier (a DOI or accession number) can improve reproducibility and make it easier for other scientists to expand on your work. Papers with open data are cited more often than papers without open data. Some institutions have an expert who can provide advice on data sharing.
?	Self-acknowledged limitations	ℹ An explicit section about the limitations of this study was not found. We encourage authors to include a paragraph in the discussion that addresses study limitations. Every study has limitations. Describing these limitations helps readers to understand and contextualize the research.
✓	Conflict of interest statement	ℹ Thank you for stating any conflicts of interest.
✓	Funding statement	ℹ Thank you for reporting your funding source(s), or stating that there was no specific funding for this work.
n/a	Registration statement	ℹ A registered protocol makes the planned research project more transparent and can protect against bias. Please consider registering future confirmatory studies.

Generated by ODDPub, limitation-recognizer, rtransparent, trial-identifier.

Figures

✓	No bar graphs of continuous data	ℹ Thank you for not using bar graphs to present continuous data. Many different datasets and data distributions can lead to the same bar graph, and the actual data may suggest different conclusions from the summary statistics alone.
?	Rainbow color maps	ℹ Please consider replacing the rainbow ("jet") colormap(s) that we detected in at least one figure with alternatives like Viridis or Cividis. Rainbow color maps distort readers' perception of the data by introducing visual artifacts. Rainbow color maps are also inaccessible to readers with colorblindness. Alternatives colormaps like Viridis and Cividis are accessible to colorblind readers and avoid visual artifacts.

Generated by JetFighter, Barzooka.
