# Supplementary material for: Deciphering intercellular communication in the cerebellar external granule layer: Insights into non-classical connections in neural development
Source: PLoS One. 2026 Jun 25;21(6):e0352088. doi: 10.1371/journal.pone.0352088 (PMC13298772; doi:10.1371/journal.pone.0352088)
Supplement: Peer review history

# Author comment: Author response to Decision Letter 0

**DOI:** 10.1371/journal.pone.0352088.r001

4 Apr 2026

Attachment Submitted filename: full-revision_j.pdf

# Peer review: Decision Letter 0

**DOI:** 10.1371/journal.pone.0352088.r002

**Authors:** Maria Teresa Fiorenza

5 Jun 2026

Deciphering Intercellular Communication in the Cerebellar External Granule Layer: Insights into Non-Classical Connections in Neural Development

PONE-D-26-16284

Dear Dr. Zurzolo,

We’re pleased to inform you that your manuscript has been judged scientifically suitable for publication and will be formally accepted for publication once it meets all outstanding technical requirements.

Within one week, you’ll receive an e-mail detailing the required amendments. When these have been addressed, you’ll receive a formal acceptance letter and your manuscript will be scheduled for publication.

An invoice will be generated when your article is formally accepted. Please note, if your institution has a publishing partnership with PLOS and your article meets the relevant criteria, all or part of your publication costs will be covered. Please make sure your user information is up-to-date by logging into Editorial Manager at Editorial Manager® and clicking the ‘Update My Information' link at the top of the page. For questions related to billing, please contact billing support.

If your institution or institutions have a press office, please notify them about your upcoming paper to help maximize its impact. If they’ll be preparing press materials, please inform our press team as soon as possible -- no later than 48 hours after receiving the formal acceptance. Your manuscript will remain under strict press embargo until 2 pm Eastern Time on the date of publication. For more information, please contact onepress@plos.org.

Kind regards,

Maria Teresa Fiorenza

Academic Editor

PLOS One

Journal requirements:

When submitting your revision, we need you to address these additional requirements.

1. Please ensure that your manuscript meets PLOS ONE's style requirements, including those for file naming. The PLOS ONE style templates can be found at

https://journals.plos.org/plosone/s/file?id=wjVg/PLOSOne_formatting_sample_main_body.pdf and

https://journals.plos.org/plosone/s/file?id=ba62/PLOSOne_formatting_sample_title_authors_affiliations.pdf

2. Please update your submission to use the PLOS LaTeX template. The template and more information on our requirements for LaTeX submissions can be found at http://journals.plos.org/plosone/s/latex.

3. Thank you for stating the following financial disclosure:

“Institut Pasteur (Pasteur Institute): Chiara Zurzolo, Big Brain Theory Program; Institut du Cerveau et de la Moelle Epinière (ICM): Laurence Cathala, Big Brain Theory Program; Agence Nationale de la Recherche (ANR): Chiara Zurzolo, ANR-16-CONV-0005; Fondation pour la Recherche Médicale (FRM): Chiara Zurzolo, EQU202103012630; Agence Nationale de la Recherche (ANR): Chiara Zurzolo, AAPG2023 UnProSec 2024-2027; Fondation pour la Recherche Médicale (FRM): Chiara Zurzolo, MND202310017892; Conseil régional d'Ile-de-France: Gabriel Kaddour; DIM ELICIT: Gabriel Kaddour; Fondation pour la Recherche Médicale (FRM): Gabriel Kaddour, Financement de Fin de thèse”

Please state what role the funders took in the study.  If the funders had no role, please state: "The funders had no role in study design, data collection and analysis, decision to publish, or preparation of the manuscript."

If this statement is not correct you must amend it as needed.

Please respond by return e-mail so that we can amend your financial disclosure and competing interests on your behalf.

Additional Editor Comments:

This manuscript investigates the presence and nature of intercellular communications in the EGL of the developing cerebellum, an ideal system for this purpose, as it is populated by proliferating progenitors, post-mitotic neurons, and migrating neurons. The study is conceived as an extension of an earlier one to properly characterize “non-canonical” connections, including tunneling nanotubes (TNTs).

By using a combination of sparse genetic labeling, live imaging, and automated CNN-based segmentation to study delicate and transient structures that are usually challenging to preserve and identify in vivo, the study goes beyond in vitro models to identify and distinguish various types of intercellular connections (ICs) in a highly dynamic mammalian tissue environment. When measuring cell populations and connections, this multimodal technique ensures high technical precision.

The key finding is that not all reported connections can be explained by cytokinetic bridges (CBs), and that cell-to-cell connections during brain development may include a different form of communication called TNT-like connections. In particular, clonally linked (87%) and non-clonally related (13%) cells appear to generate TNT-like connections de novo, offering a nuanced perspective on how these structures might promote communication across various cell lineages.

Despite a thorough and precise description, some questions remain about the nature and function of these novel communication modalities. The term "TNT-like," rather than definitive identification, results from the authors' reliance on "negative controls"—the absence of CB/IB markers—and morphological criteria, given the lack of molecular markers for TNTs. Accordingly, the absence of conclusive evidence of cargo transfer (such as organelle or protein exchange) via these structures does not rule out the possibility that some structures are only closed-ended filopodia or cytonemes. These limitations persist even after the authors' thorough and careful revision, primarily due to the technical constraints of live imaging. For instance, attempts to detect cytoplasmic transfer using photoconvertible proteins (Dendra2) failed due to tissue density and light refraction, casting doubt on the "open-ended" nature of these connections, as suggested by earlier EM evidence. Advances in presently available methodologies will make this type of research easier.

The study is deemed worthy of publication because it: i) is methodologically sound and correct and offers a rigorous interpretation of the results without overstatements; ii) adds a novel insight about cell interactions that likely control cell fate during neural development, which can help a better understanding of neurodevelopmental disorders, among other things.

I suggest the following small change:

- Introduction, line 49: the term “pathway” is not appropriate here, as the authors refer to cell-to-cell communication modalities.

Reviewers' comments:

Reviewer's Responses to Questions

**Comments to the Author** 

1. Is the manuscript technically sound, and do the data support the conclusions?

The manuscript must describe a technically sound piece of scientific research with data that supports the conclusions. Experiments must have been conducted rigorously, with appropriate controls, replication, and sample sizes. The conclusions must be drawn appropriately based on the data presented.

Reviewer #1: Yes

Reviewer #2: Yes

**********

2. Has the statistical analysis been performed appropriately and rigorously?

Reviewer #1: Yes

Reviewer #2: Yes

**********

3. Have the authors made all data underlying the findings in their manuscript fully available?

The PLOS Data policy requires authors to make all data underlying the findings described in their manuscript fully available without restriction, with rare exception (please refer to the Data Availability Statement in the manuscript PDF file). The data should be provided as part of the manuscript or its supporting information, or deposited to a public repository. For example, in addition to summary statistics, the data points behind means, medians and variance measures should be available. If there are restrictions on publicly sharing data—e.g. participant privacy or use of data from a third party—those must be specified.

Reviewer #1: Yes

Reviewer #2: Yes

**********

4. Is the manuscript presented in an intelligible fashion and written in standard English?

PLOS ONE does not copyedit accepted manuscripts, so the language in submitted articles must be clear, correct, and unambiguous. Any typographical or grammatical errors should be corrected at revision, so please note any specific errors here.

Reviewer #1: Yes

Reviewer #2: Yes

**********

5. Review Comments to the Author

Please use the space provided to explain your answers to the questions above. You may also include additional comments for the author, including concerns about dual publication, research ethics, or publication ethics. (Please upload your review as an attachment if it exceeds 20,000 characters)

Reviewer #1: This is an automated report for PONE-D-26-16284. This report was solicited by the PLOS One editorial team and provided by ScreenIT.

ScreenIT is an independent group of scientists developing automated tools that analyze academic papers. A set of automated tools screened your submitted manuscript and provided the report below. Each tool was created by your academic colleagues with the goal of helping authors. The tools look for factors that are important for transparency, rigor and reproducibility, and we hope that the report might help you to improve reporting in your manuscript. Within the report you will find links to more information about the items that the tools check. These links include helpful papers, websites, or videos that explain why the item is important. While our screening tools aim to improve and maintain quality standards they may, on occasion, miss nuances specific to your study type or flag something incorrectly. Each tool has limitations that are described on the ScreenIT website. The tools screen the main file for the paper; they are not able to screen supplements stored in separate files. Please note that the Academic Editor had access to these comments while making a decision on your manuscript. The Academic Editor may ask that issues flagged in this report be addressed. If you would like to provide feedback on the ScreenIT tool, please email the team at ScreenIt@bih-charite.de. If you have questions or concerns about the review process, please contact the PLOS One office at plosone@plos.org.

Reviewer #2: It is a pleasure to review this excellently revised paper which has responded to thorough reviews by 3 recent reviewers and was extensively revised to meet their criticisms/suggestions. For this reviewer, it is an exciting paper because it presents methods, and demonstrates their use, to study developing tunneling nanotubes (TNTs) in living samples of mouse pups. The use of electroporation to introduce drug-controlled constructs only expressed in neural precursors, into the developing cerebellum allows tissue to be collected and studied in organ culture for short periods. The use of a variety of differently-labelled antibodies to detect mitotic stages and distinguish intercellular bridges remaining from cytokinesis from TNTs, has allowed a method to watch them grow. While some of these may be cytonemes capable of cargo transfer, v,s, TNTs with signaling capabilities, further studies will clarify this point. This is a great advance from the use of scanning em which only allows static views (fixatives for the usual light microscopy mostly destroy them).

**********

6. PLOS authors have the option to publish the peer review history of their article (what does this mean?). If published, this will include your full peer review and any attached files.

If you choose “no”, your identity will remain anonymous but your review may still be made public.

**Do you want your identity to be public for this peer review?**      For information about this choice, including consent withdrawal, please see our Privacy Policy.

Reviewer #1: No

Reviewer #2: No

**********

Attachment Submitted filename: report_10.1101+2025.08.11.669634.pdf

# Editor report: Acceptance letter

**DOI:** 10.1371/journal.pone.0352088.r003

**Authors:** Maria Teresa Fiorenza

PONE-D-26-16284

PLOS One

Dear Dr. Zurzolo,

I'm pleased to inform you that your manuscript has been deemed suitable for publication in PLOS One. Congratulations! Your manuscript is now being handed over to our production team.

At this stage, our production department will prepare your paper for publication. This includes ensuring the following:

* All references, tables, and figures are properly cited

* All relevant supporting information is included in the manuscript submission,

* There are no issues that prevent the paper from being properly typeset

You will receive further instructions from the production team, including instructions on how to review your proof when it is ready. Please keep in mind that we are working through a large volume of accepted articles, so please give us a few days to review your paper and let you know the next and final steps.

Lastly, if your institution or institutions have a press office, please let them know about your upcoming paper now to help maximize its impact. If they'll be preparing press materials, please inform our press team within the next 48 hours. Your manuscript will remain under strict press embargo until 2 pm Eastern Time on the date of publication. For more information, please contact onepress@plos.org.

You will receive an invoice from PLOS for your publication fee after your manuscript has reached the completed accept phase. If you receive an email requesting payment before acceptance or for any other service, this may be a phishing scheme. Learn how to identify phishing emails and protect your accounts at https://explore.plos.org/phishing.

If we can help with anything else, please email us at customercare@plos.org.

Thank you for submitting your work to PLOS ONE and supporting open access.

Kind regards,

PLOS ONE Editorial Office Staff

on behalf of

Prof. Maria Teresa Fiorenza

Academic Editor

PLOS One
